# Early onset children’s Gitelman syndrome with severe hypokalaemia: a case report

**DOI:** 10.1186/s12887-020-02265-9

**Published:** 2020-08-05

**Authors:** Hanjiang Chen, Rong Ma, Hongzhe Du, Jin Liu, Li Jin

**Affiliations:** Department of Paediatrics, First Teaching Hospital of Tianjin University of Traditional Chinese Medicine, 88 Changling Road, Xiqing district, Tianjin, 300000 China

**Keywords:** Gitelman syndrome, Severe hypokalaemia, Early onset, SLC12A3

## Abstract

**Background:**

Hypokalaemia is a common condition among paediatric patients, but severe hypokalaemia is rare and can be life-threatening if not treated properly. The causes of hypokalaemia are complex. Finding the root cause is the key.

**Case presentation:**

This article reports on a 2-year-old boy with severe hypokalaemia who was diagnosed with pneumonia. The child’s lab findings were low blood potassium minimum level of 1.7 mmol/L, hypomagnesemia, and metabolic alkalosis. However, he was without the common features of hypokalaemia, such as respiratory paralysis, severe arrhythmia, weakness and decreased blood pressure. After recovering from pneumonia, his potassium levels did not return to normal. This outcome was suspected to be due to chronic renal loss of potassium. After undergoing second-generation gene sequencing tests, it was discovered he carried the SLC12A3 gene mutation with an Asp486Asn mutation site, which he had inherited from his mother. The final diagnosis was made, confirming the child suffered from Gitelman syndrome.

**Conclusions:**

Genetic predisposition is an important cause of hypokalaemia in children. Children with unexplained persistent hypokalaemia should be examined for the possibility of Gitelman syndrome, which should be distinguished from Bartter syndrome. Genetic testing is the gold standard.

## Background

Gitelman syndrome (GS) is a rare autosomal recessive renal disorder [1]. GS is caused by mutation of the SLC12A3 gene. This gene is responsible for the thiazide diuretic-sensitive sodium chloride co-transporter (NCCT) located in the renal distal convoluted tubule of the kidney. Mutations of this gene result in structural or functional abnormalities in the NCCT, preventing normal absorption of sodium chloride in the renal distal convoluted tubule. Most children only show non-specific symptoms such as fatigue, thirst, and polyuria; a few show complications such as developmental retardation, convulsions, and rhabdomyolysis [2]. Based on the benign progression of GS, the disease is most commonly diagnosed during adulthood, so the incidence of infants and young children is rare [3]. At the same time, infants and young children with hereditary hypokalaemia need to be distinguished from those with Barter syndrome (BS) (see Table 3 for details). BS commonly manifests with the same symptoms of renal potassium loss, low chloride and metabolic alkalosis. The most significant differences between them are hypomagnesemia, low urine calcium and genetic testing, which is the gold standard. This article reports on an early-onset case of GS, a case that includes severe hypokalaemia and its genetic phenotype and electrolyte changes.

## Case presentation

A male patient, 2 years old, was admitted to the hospital on May 21, 2018 due a sustained fever of over 6 consecutive days, with his highest body temperature reaching 39.0 °C, which peaked once or twice per day, accompanied by coughing, phlegm, and shortness of breath. His local hospital diagnosed him with “acute upper respiratory tract infection” and prescribed him 5 days of Chinese herb medicine; however, his temperature was not alleviated. After entering our hospital, his chest X-ray showed that both of his lungs had an increased thickened texture. With possible inflammation suspected, the boy was then admitted as a pneumonia patient. Prior to the onset of the illness, the child’s spirit was normal, with no irritability or fatigue. His dietary intake was also normal, with normal appearing defecation. His medical history showed that he was a rather healthy baby, G1P1 (Gravida 1, Para 1) full-term delivery. He was breastfed and had normal growth and development for his age, and his parents were also healthy. As a child, he had no history of food or drug allergies reported, and no oral diuretics or catharsis drugs were taken previously. However, the child had a history of spontaneous night-sweats and enuresis according to his parents.

### Physical examination

Body temperature 37.0 °C, pulse 125 beats/min, breathing 25 breaths/min, blood pressure 95/65 mmHg, weight 10.5 kg, height 92 cm, slightly underweight (boy standard weight: 11.2–14.0 kg). Normal reflexes without shortness of breath or cyanosis. No rash, no swelling of superficial lymph nodes, pharyngeal hyperaemia. Bilateral tonsils were not enlarged. Rough tracheal sounds with phlegm rales were heard. Heart and abdominal examinations were normal. Extremities and spine were normal, physiological reflexes existed, and pathological reflexes were not elicited.

### Auxiliary examination

Blood test showed WBC 14.85 × 10^9^/L, N% 78.2, L% 14.6, HGB141 g/L, PLT 290 × 10^9^/L, CRP10.0 mg/L. Stool and urine routines were normal; procalcitonin 0.3 ng/L; ESR 15 mm/h; ferritin 90.8 ng/ml; ASO normal; Mycoplasma pneumoniae antibody IgM negative. Liver and kidney function, glucose, coagulation function, rheumatoid index, thyroid function, lymphocyte subtype tests (NK cells, T cells, auxiliary T cells, reactive T cells, B cells) and immunoglobulins all met the reference ranges of his age group; repeated examinations of electrolytes indicated hypokalaemia, hypomagnesemia, low chlorine, low sodium and transient mild metabolic alkalosis (see Table 1 for details). A further check of the 24-h urinary potassium was 57 mmol/24 h, and the 24-h urinary calcium was 2.86 mmol/24 h, which informed increased urinary potassium level; plasma renin activity was 142.05 pg/ml (4–24 pg/ml); angiotensin II was 435.62 pg/ml (25–129 pg/ml); aldosterone was 100.26 pg/ml (10–160 pg/ml); and serum cortisol and adrenocorticotropic hormone were normal. Multiple reviews of ECG and 24-h Holter showed 1st degree atrioventricular block (see Table 2 for details), cardiac colour Doppler showed tricuspid valve, mild regurgitation of the pulmonary valve; abdominal colour Doppler showed intrahepatic calcification plaque and accumulation of gas in the colon; renal colour Doppler ultrasound and adrenal colour Doppler ultrasound did not appear abnormal.

**Table 1:** Serum electrolyte changes over 7 days of hospitalization

	Day 1	Day 1 (8 h later)	Day 2	Day 3	Day 4	Day 5	Day 7
Ph	7.47					7.4	
k + (mmol/L)	1.7	2.1	2.6	3.1	3.1	2.87	3
Na + (mmol/L)	132.3	136.5	134.3	136.8	137.7	141	140.5
cCa2 + (mmol/L)	1.05			2.23		1.25	
Mg2 + (mmol/L)	0.59			0.43		0.6	
HCO3-	23.82	27.11	23.03	23.28	21.35	25	21.83
Cl-(mmol/L)	93.3	97.2	97.4	99.5	100.5	102	103
OSM(m OSM/L)	272.94	280.57	275.51	280.2	282.85	290.01	290.01

The blood samples of days 1 and 5 are biochemical items; the other days’ blood samples are seven emergencies (including K, Na, Cl, CO2CP, Glu, BUN, Cr, AG, and OSM)

**Table 2:** Correlation between EKG and serum electrolytes

	Heart rate (min)	PR interval (ms)	QT interval (ms)	K+ (mmol/L)	Mg2+ (mmol/L)
Day 1	127	208	420	1.7	0.59
Day 2	106	196	470	2.6	
Day 3	129	162	298	3.1	0.43
Day 4	115	172	296	3.1	
Day 5	102	174	324	2.87	0.6
Day 6	83	192	350		
Day 7	92	202	344	3	0.6

### Diagnosis and treatment

**Fig. 1:**
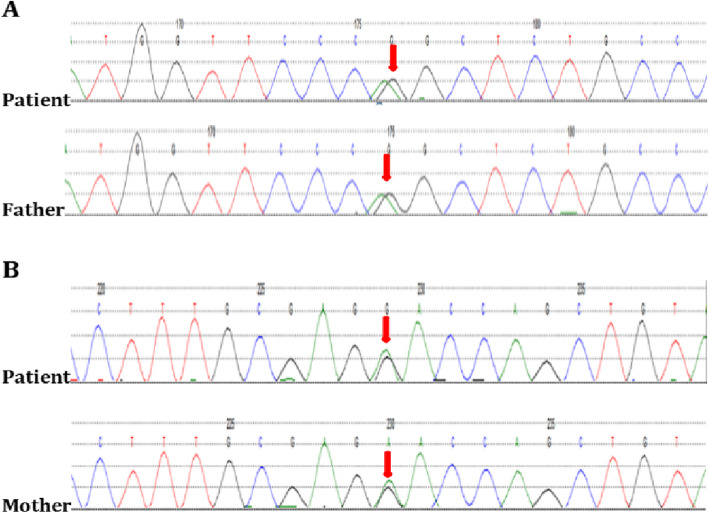
Sanger sequencing of candidate variants. **a** Gene location (No. Intron 12, cDNA level 602-16G > A) is a suspicious disease-causing gene derived from the father. **b** Gene location (exon number Exon 12, cDNA level 1456G > A, protein level Asp486Asn), considered to be the disease-causing gene from the mother

**Fig. 2:**
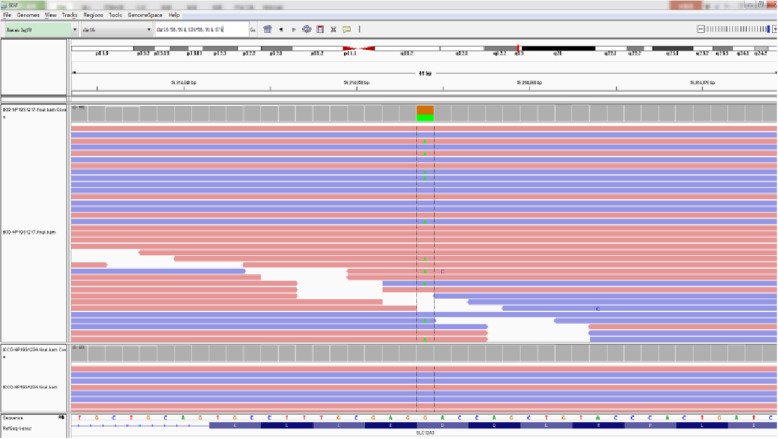
Integrative Genomics Viewer (IGV) snapshot of the sequence data at SLC12A3 for the child’s sample and the corresponding calls made from the data

According to the symptoms, signs and chest radiographs of the child, he could be diagnosed with pneumonia. Analysis of pathogens and blood test results showed a high total number of white blood cells. Neutrophils were dominant, along with high CRP and PCT. According to the infection index combined with our clinical experience, these findings indicate the high possibility of bacterial infection. At the beginning of the treatment, intravenous ceftriaxone (80 mg/kg) was prescribed to treat the infection and to suppress coughing and phlegm with airway management. His body temperature dropped to normal within 24 h, which suggested the antibiotic was effective. On the day of admission, the emergency reports showed electrolyte values with “blood potassium 1.7 mmol/L” and “blood magnesium 0.59 mmol/L”. First, we considered the possibility of electrolyte imbalance secondary to pneumonia; thus, oral and intravenous potassium supplementation (4–5 g/day) were prescribed along with injection of magnesium sulphate and rehydration treatment. Regular review of electrolytes showed that blood potassium gradually increased to 3.1 mmol/L within 2–3 days; however, the result could not be sustained, and it was difficult to make it continue to rise. From further questioning of the child’s medical history according to the parents, it was learned that the child had long-term “sleepiness,” “drowsiness” and other symptoms accompanied by enuresis (3–4 times/week) and severe night sweats (parents described it as “like a shower”); he was also slightly light in weight. All of the above could have been due to possible chronic potassium loss. With persistent hypokalaemia and hypomagnesemia observed in the clinical stay, his urinary potassium excretion increased, and his urinary calcium was normal. The blood biochemical analysis showed that the PH and HCO3- were high. His blood pressure was normal, but plasma renin activity and angiotensin were high. The aldosterone levels were normal, and the clinical consideration of “Gitelman syndrome” was likely. Further second-generation gene analysis (KingMed Diagnostics) (see Figs. 1 and 2 for details) detected two heterozygous mutations (SLC12A3 (16q13/NM-000339.2)): exon number Exon12, cDNA level 1456G > A, protein level Asp486Asn, considering that the disease gene was derived from the mother; and exon No. Intron12, cDNA level 602-16G > A, protein levels were normal, considering a suspicious pathogenicity derived from the father. With the Gitelman syndrome diagnosis having been established, long-term oral potassium citrate granules were prescribed to be taken for 1 year. Blood potassium levels were stable between 3.1–3.5 mmol/L, and the PR intervals were between 0.17–0.2 cm. Growth and development were normal, and the enuresis disappeared, but the night sweats still persisted.

## Discussion and conclusions

GS is an autosomal recessive disorder caused by mutations in the SCL12A3 gene, which encodes a thiazide-sensitive sodium chloride co-transporter. Most cases that develop symptoms are in adolescents or adults; the disease is less common in young children. This case was diagnosed in early childhood and was induced after infection. The incidence of this disease is low. Once considered a rare disease, with the advancement of genetic technology, clinical reports have increased [4]. In the current study of GS, mainly consisting of case reports, there has been little analysis of the disease. Searching through nearly 30 years of literature resulted in only 6 cases of large-scale disease research. These included 3 articles in China (sample size is 67, 47, 20 people); 1 article in Switzerland [5] (163 people), 1 in the UK [6] (36 people), and 1 in South Korea [7] (34 people). The remainders of the relevant research were mainly case reports reflecting the complications reported, such as combined Sjogren’s syndrome [8], combined pregnancy or pregnancy HELLP syndrome [9], primary hyperparathyroidism [10], complete growth hormone deficiency [11], schizophrenic changes [12], and Fanconi syndrome [13]. Hypokalaemia is a common significant feature among them.

In children with hereditary hypokalaemia, GS should be distinguished from BS.BS is also an inherited renal tubular disease characterized by hypokalemia, metabolic alkalosis, and secondary RAA system activation. The pathological basis is that the CLCNKB gene mutation in the thick segment of the ascending branch of the loop of Henle leads to Cl-resorption disorder. BS mainly occurs in children, and more than half of the patients occur in children under 5 years old.BS is categorized into neonatal type, classic type and variant type (that is, Gitelman syndrome) according to the day of onset. Therefore, GS was once considered to be a variant of BS. However, the genetic basis of the two is different, and the differences are also reflected in the clinical manifestations, biochemistry evolution, treatment and prognosis (see Table 3 for details). Blood magnesium levels, 24-h urinary calcium and gene loci are the keys to identification. The pathological basis of GS is in the renal distal tubule. BS is located in the medullary thick ascending limb, and the adjustment and balance of magnesium ion in the body occurs in the distal tubule. The reabsorption of the basal cells of the distal tubule determines the final excretion of magnesium ions. However, GS with normal blood magnesium and urinary calcium also exists clinically; thus, only genetic testing is the golden indicator. The clinical phenotype is related to the genetic phenotype. GS is generally considered to occur in adolescents or adults and is rare in infants and young children. The child’s weight is on the low side of the normal range, the height is within the normal range, and metabolic alkalosis and RAS system activation are not obvious and are likely to be related to clinical phenotypes. The condition is also associated with the treatment of pneumonia, IV fluid infusion, and correction of electrolyte disturbances. At the same time, GS is a rare disease, its awareness is lacking in the early stage of disease treatment, and the relevant tests are not perfect; however, fortunately, we performed a genetic test in time to clarify the diagnosis.

**Table 3:** Differences between Gitelman syndrome and classic Bartter syndrome

	Gitelman syndrome	Bartter syndrome
Time	Adolescent or adult	Childhood
Hypokalaemia	yes	yes
Hypochloric metabolic alkalosis	yes	yes
High renin activity	yes	yes
Hypomagnesemia	yes	no
Urinary calcium	low	low, normal or hypercalciuria
Development retardation	rare	yes
Location	renal distal tubule	medullary thick ascending limb
Gene mutation	SLC12A3	CLCNKB

Diagnosis of the disease is mainly based on the SCL12A3 gene. At present, more than 400 genetic mutations have been identified worldwide, among which missense mutations are the most common. A total of 18% are homozygous mutations, more than 45% are compound heterozygous mutations, and 7% of patients have more than 3 mutations [14]. However, the number of mutations is not positively correlated with clinical manifestations and symptoms. The mutation sites in different regions are different. The IVS9 + 1G > T shear mutation is the most common in Europeans, and the T60M site mutation is more common in Chinese-related reports [15]. Up to now, more than 100 mutant genes have been found in the Chinese study of GS [16]. The mutation site of this patient was Asp486Asn, which belongs to the missense heterozygous mutation. The amino acid of the 486th protein was changed from Asp to Asn, and the causative gene was derived from the mother. Detection of this mutation has been reported in the literature. As the popular mutation sites have not been reported in younger children, this article enriches the clinical and genetic characteristics of GS in younger children. Currently, there are no systematic reports on the studies of GS based on the correlations among GS, electrolyte and EKG changes. Hypokalaemia is an inevitable characteristic, but the severity is different. There was no corresponding relationship between the length of the PR interval and the blood potassium concentration. The PR interval was not shortened after the hypokalaemia was corrected. The internal mechanism may be related to the gene itself, and long-term follow-up is also necessary. GS is generally benign, and its severity is not positively correlated with blood potassium levels. We will have a long-term follow-up with this child, and further assessment of the prognosis is needed.

## Data Availability

All data generated or analysed during this study are included in this published article.

## Abbreviations

GS: Gitelman syndrome
BS: Bartter syndrome
WBC: White blood cells
N%: Neutrophil ratio
L%: Lymphocyte ratio
HGB: Haemoglobin
PLT: Platelets
CRP: C-reactive protein
ESR: Erythrocyte sedimentation rate
ASO: Anti-streptolysin O test
